# Cancer, Trichinosis and Tuberculosis—Are They Incurable?

**Published:** 1881-01

**Authors:** J. G. Westmoreland

**Affiliations:** Atlanta, Ga.


### CANCER, TRICHINOSIS AND TUBERCULOSIS—ARE THEY INCURABLE?

By J. G. WESTMORELAND, M.D., Atlanta, Ga.

Diseases considered necessarily fatal are treated with reference alone to the comfort of the patient. Medicine being a progressive, not a perfect science, physicians should not content themselves with knowing and practicing upon the discoveries of their predecessors. Scrofula was once considered incurable, and the unfortunate subject was, by the ancients, doomed to certain death after a short disgusting life of misery. At this day, the physician who would seek nothing more than the ease and comfort of his scrofulous patient would not be considered as having been educated in the general principles of his profession. To the investigating energy and experiment of medical men, the world is now indebted for a cure of this otherwise fatal disease. Additions in therapeutics are often made by accident, but members of the medical profession should not wait for this. The action of certain remedies being known beyond dispute, their application can be made, by the exercise of reason, to the successful treatment of diseases in which they had not before been used. By accident or otherwise, iodine was found to be a curatine remedy for scrofula and goiter. No longer are the loathsome subjects and victims of these diseases suffered to take paliative treatment alone.

In the successful treatment of such diseases with iodine, investigation sprang up as to the physiological action of the remedy in producing such wonderful result. At first the action was called "alterative," and for a long time remedies which were found effective in the treatment of various chronic ailments were associated under this name. The articles used in syphilis, scrofula, goiter, torpid liver, etc , were called alteratives, because, by some unknown action or process, a change or alteration was had. If we should call this action catalytic, eliminative or destructive of the virus or substance upon which the diseased development depends, it is easy to explain the manner of relief, and to experiment with the remedies in the treatment of other diseases dependent upon matters to be eliminated.

It has been ascertained that iodine not only has this action upon morbific substances, circulating in the blood and deposited in soft structures, but that a favorable local action is had upon chronic inflammation and ulceration, from whatever cause. This has been termed the "local alterative" action. Now, this remedy, it would seem, is peculiarly suited, not only to the removal or elimination of the morbific agent, but to the cure of local disturbances produced by it. Iodine introduced into the blood, eliminates the morbific agents in syphilis and scrofula, and by local application hastens the cure of ulcers produced by it. It, then, has catheretic or local alterative action and general alterative action. Shall we content ourselves with the discoveries of others in the use of this valuable remedy? We can now more safely experiment since the discoveries already made, and if a thorough test should disclose the fact that tuberculous matter like scrofulous can also be eliminated, and its further production prevented, a still greater boon to suffering humanity will be afforded. Already I have satisfied myself, beyond a doubt, that the local effects of tuberciles upon the respratory mucous membrane, and in the vescular structure of the lungs can be perfectly relieved by the local alterative or catheretic action of the remedy. In fact, no other remedy so far as I know, can be applied directly to these parts in sufficient concentration or strength, to make decided impression. No other known to be catheretic in action is subject to the form of fumes of sufficient permanence, to reach the whole respiratory apparatus.

Now let a thorough test be made with iodine in tuberculosis, in proper doses for its introduction into the circulation as in scrofula. Reasoning from analogy, there seems to be a possibility of success. The two diseases are developed in the same diathesis, and the same depressing influences are necessary to the development of each. The strumous constitution exists in both, and although the scrofulous matter is deposited generally in different organs from that of tuberculosis, it is not unreasonable to expect the latter substance also to be eliminated by iodine. Should this be proven by faithful experiment, which certainly can be had safely to the patient, then we will have in the same remedy that which will eliminate the morbific matter and cure the local disease in the respiratory organs made by it. Inhalation is not sufficient for this purpose. Although some absorption may be had in this way, not enough will be received into the blood to test its catalytic action upon tubercles. My plan of inhaling for bronchial and pulmonary chronic irritation or ulceration produced by tubercles, or other cause, is to place in a drachm morphine vial, previously heated on a stove or over a lamp, a small particle, say half a grain, of iodine and have the patient to inhale the fumes by the mouth or through the nostrils. This should be repeated every third or fourth day, and sometimes more frequently, preceded by a dose of morphine if much cough be produced by it. This remedy as a catalytic in scrofula, syphilis or toberculosis is required in much larger quantity and repeated three times a day.

Experiments in the treatment of tuberculosis with the hypophosphites, jaborandi, etc., for a time promised favorable results, but have not given confidence in the remedies. Cod liver oil is retained in the prescriptions of most physicians, and properly so, no doubt, not however as a medicinal agent, but as fatty food, which must be admitted as important in keeping up the necessary heat and vigor of strumous subjects.

Cancer, another of the so called incurable diseases, dependent on a morbific element, germ or cell, developes destructive ulceration of structures. Various excharotic nostrums have been used by empirics for the destruction of tissue connected with malignant and non-malignant tumors and ulcers, but medical men consider the knife preferable, of course, for the mere removal of structures thus affected, rather than this slow painful process. Arsenic, one of the escharotic ingredients, was for a time considered catalytic of the cancer cell, or germ, and hence given in full doses for months in order to eradicate the disease from the system. This had its day, and now Chian turpentine is exciting the profession with the prospect of a cure for this fatal malady. By all means let a thorough test be made, and if it proves to be a catalytic of the fatal element, let the world rejoice at this successful attempt to relieve suffering humanity. I must insist, however, on the treatment of cancerous ulcers, wherever located, with carbolic acid. Attention has already been called to the subject in this journal, and after experiments with it which authorized confidence in the remedy. My experiments in future will be directed not only with a view of arresting the progress of ulceration by the local application of the acid, but also to test its eliminative or catalytic powers. The use of three or four grains internally, three times a day, while the application of the pure crystals, dissolved by heat, is made to the ulcer every third or fourth day, will not prove a dangerous experiment, and hopes are entertained of great benefit from it.

With the fatal disease produced by the trichina spiralis, few physicians have any experience, and those who have met with the unfortunate subject are scarcely more informed on the subject of cure. When innumerable small animals are consuming all muscular structures, their destruction must be effected in order to safety of the subject. So far as is generally known no destroyer of the animal has been discovered. An effort in this direction should certainly be made. Is it possible to reach the animal by any poison, if we knew what would act as a poison upon it? Reasoning on the subject, brings us to the conclusion that, mercury which acts as a poison to all parisites, to which we have ever been able to apply it, may also prove destructive to trichinæ. In order to reach it, the remedy must find its way to every part of the body, and this can be effected only through the circulation. If then any preparation of mercury be administered so as to get into the circulation, the greatest amount possible without serious injury to the patient, enough may find its way into the tissues inhabited by the trichenæ to produce their destruction. In the absence of any known remedy for this fatal disorder, by all means a test should be made of the effects of mercury administered to the full extent allowable, internally and by inunction.

